# Technological Aspects and Evaluation Methods for Polymer Matrices as Dental Drug Carriers

**DOI:** 10.3390/biomedicines11051274

**Published:** 2023-04-25

**Authors:** Dorota Kida, Tomasz Konopka, Kamil Jurczyszyn, Bożena Karolewicz

**Affiliations:** 1 Department of Drug Form Technology, Wroclaw Medical University, Borowska 211 A, 50-556 Wroclaw, Poland; bozena.karolewicz@umw.edu.pl; 2 Department of Periodontology, Wroclaw Medical University, Krakowska 26, 50-425 Wroclaw, Poland; 3 Department of Dental Surgery, Faculty of Medicine and Dentistry, Medical University of Wroclaw, Krakowska 26, 50-425 Wroclaw, Poland

**Keywords:** dental applications, dental carriers, freeze-casting method, property evaluation methods, solvent-casting method, technological parameters, 3D printing

## Abstract

The development of polymer matrices as dental drug carriers takes into account the following technological aspects of the developed formulations: the composition and the technology used to manufacture them, which affect the properties of the carriers, as well as the testing methods for assessing their behavior at application sites. The first part of this paper characterizes the methods for fabricating dental drug carriers, i.e., the solvent-casting method (SCM), lyophilization method (LM), electrospinning (ES) and 3D printing (3DP), describing the selection of technological parameters and pointing out both the advantages of using the mentioned methods and their limitations. The second part of this paper describes testing methods to study the formulation properties, including their physical and chemical, pharmaceutical, biological and in vivo evaluation. Comprehensive in vitro evaluation of carrier properties permits optimization of formulation parameters to achieve prolonged retention time in the dynamic oral environment and is essential for explaining carrier behavior during clinical evaluation, consequently enabling the selection of the optimal formulation for oral application.

## 1. Introduction

When designing a drug formulation for use in dentistry, it is necessary to consider aspects of the application of the carrier in the oral cavity, which is a diverse area and includes the lips, cheeks, the dorsal and lower surface of the tongue, hard palate, soft palate, maxillary and mandibular alveolar processes (gums), and the floor of the mouth, covered by a mucous membrane that is constantly moistened and flushed with saliva [1]. When developing therapeutic formulations for topical application in the oral cavity, it is necessary to take into account the leaching of the drug carrier by saliva, the poor penetration of the drug into the tissue, the limited adhesion surface of the preparation, its taste, the possibility of accidental ingestion of the carrier, and the aforementioned specificity of the site of application, or the disease entity being treated, i.e., therapy for lesions involving more superficial layers of epithelium, e.g., therapy for pseudomembranous candidiasis, or therapy for lesions requiring deeper penetration of the substance, e.g., therapy for Wilson’s lichen and other mucocutaneous diseases [1,2,3,4,5,6,7]. In addition, there is the need for direct administration of the drug into periodontal pockets when supplemented mechanical therapy pocket cleaning is indicated [2]. Regardless of the type of carrier and the site of application in the oral cavity, dentists point to the desirable properties of the administered form of the drug, i.e., adhesion, moldability, adaptability to the application site (plasticity), resistance to smearing, modified release in the application site, penetration into tissue, ease of application including patient self-application, and biodegradability [2,3]. Answering an urgent dental clinical demand is the development of local drug-delivery carriers, i.e., different types of film, sponge, wafer, nanofiber and 3D-printed carriers that minimize systemic exposure to the drug [2,8,9,10,11,12,13,14,15,16,17] and limit potential adverse reactions [1,3,18,19]. Modern advances in the technology of drug formulations used for oral cavity application are primarily based on the use of macromolecular compounds, including those of natural origin, i.e., gelatin, sodium alginate, chitosan, xanthan gum and gellan gum; semi-synthetic polymers, i.e., cellulose derivatives; and synthetic polymers, i.e., polyvinyl alcohol, polyethylene oxide, polycaprolactone, polylactides, polyamides, acrylic acid polymers, etc. [9,20,21,22]. This review discusses the technological aspects of polymeric drug carriers and their evaluation methods. This paper outlines the main methods used to produce the aforementioned carriers, i.e., the solvent-casting method, lyophilization method, electrospinning and 3D printing. The preparation of carriers using each of these technologies requires choosing the formulation composition, including the type and amount of excipients, and selecting the process conditions for preparing the carrier. Depending on the type of carrier and the technology used to manufacture it, excipients are added during the preparation process, including the vehicles that make up the carrier structure, i.e., polymers and others; solvents; taste-masking agents; and plasticizers and cross-linking agents. When considering the choice of preparation method, the technologist analyzes the possibility of producing the intended carrier structure, the process steps and the choice of parameters affecting the stability of the active substances in the formulation and the properties of the final carrier. This review also highlights the limitations of the indicated technologies in obtaining feasible formulation types and offers examples of carriers derived using them. For a comprehensive evaluation of the formulations, a range of studies is required to describe the resulting carriers for dental applications in different areas of the oral cavity, with the identification of methods enabling their comprehensive evaluation, ensuring subsequent safety and therapeutic efficacy.

## 2. Methods for Preparing Polymeric Carriers—Technologies and Parameters

### 2.1. Solvent-Casting Method (SCM)

SCM is applied to fabricate polymeric mono- and multilayer film and fast-dissolving wafers as carriers, which can be described as thin, flexible, compact and non-porous layers of polymer with or without plasticizer [23,24,25]. In this technology, before pouring the prepared mass from one or more film-forming agents dispersed in a solvent, the active pharmaceutical ingredient (API) and selected pharmaceutical excipients are added. The most common solvent is water, but organic solvents such as acetic acid, ethanol or acetone are also applied. Bulking and taste-masking agents can be added to the prepared mass, including mannitol, sorbitol, xylitol, dextrose, fructose, plasticizers and cross-linking agents. The aforementioned plasticizers, i.e., glycerol, dibutyl phthalate and polyethene glycol, are introduced to improve the mechanical properties of the obtained films [25,26,27,28]. The use of cross-linking agents such as genipin, due to its influence on the created network density and amount of intramolecular bonds in the cross-linked polymer structure, adjusts the viscosity, swelling capacity and the course of the degradation process of the prepared film [28,29,30].

**Figure 1:**
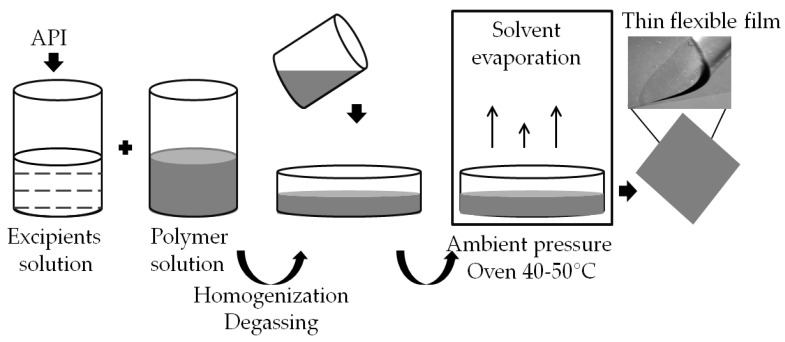
Solvent casting (SCM) steps.

In SCM, APIs and other ingredients are first dissolved in other solvent portions and combined later with polymer mass. The formulation mixing may inadvertently introduce air bubbles, so mixture deaeration is a prerequisite for a homogeneous, clear product. The trapped air is removed by centrifugation, vacuum or sonication, and the resulting solution/dispersion is cast into the mold. Finally, the solvent is allowed to evaporate at atmospheric pressure. Evaporation can be done in an oven or a dryer and on a hotplate, often at 40–50 °C, leading to faster solvent removal and forming a thin-film carrier with the drug incorporated (Figure 1). It is important to note that the cast film may retain some casting solvent after drying, contributing to its plasticization. The dried film is then cut into pieces of the desired size and adequately packaged. Finally, the resulting film carrier should not be exposed to ambient conditions for a prolonged period due to its susceptibility to drying, evaporation of the residual solvent and reduced flexibility, as well as to mechanical damage in the form of chipping or micro-cracking [31,32,33]. The quality of the final product prepared by solvent casting is influenced by the properties of the polymers used to prepare the casting compounds, such as molecule structure, transverse dimension, chain length and flexibility. With increased molecular weight and the number of polymer branches, the materials become more mechanically robust, harder, rigid and resistant to cracking [34,35].

In the solvent-casting method, when developing a reproducible product, it is important to consider and optimize the pour rate of the prepared mass, the thickness of the cast layer and the drying conditions, including temperature control, duration of this step and humidity. Advantages of this method of film preparation include uniform film thickness distribution, maximum optical purity and extremely low haze [23]. However, this process also has some limitations. For example, a thin polymer layer prepared by solvent casting becomes brittle and less flexible during storage due to evaporation or loss of residual solvent over time. Another problem associated with this method is the frequent use of organic or toxic solvents and their possible residues in the product after drying. The main limitation of this method is its unsuitability for heat-sensitive APIs. The preparation of thin films is often associated with problems from needing too long a drying time, and using a hot-air oven is not recommended for thermally sensitive drugs [24]. In addition, in the literature, the authors usually give a given temperature and drying time of the film without any control of parameters such as temperature and humidity, nor do they give the weight and surface area of the samples of individual batches. During heating, evaporation of the solvent from the upper surface of the heated mixture placed in a mold or Petri dish begins. As the solvent is removed from the top layer towards the bottom layer of the sample, the viscosity increases and the mass of the mixture changes. The drying, viscous, dense upper layer reduces/restricts the diffusion and evaporation of the solvent remaining in the lower part of the sample, which is also the case for a solvent with a lower evaporation rate. When the evaporation rate is high, rapid thickening of the evaporating layer can occur, as well as the separation of dense layers, shrinkage, precipitation of mixture components, polymers and the drug within the thickened layers, which can cause the loss of homogeneity of the sample structure. The evaporation rate depends on factors such as the humidity and temperature of the air surrounding the liquid surface, air movement, drying mass humidity, air void content, available sample area and the solvent evaporation rate. The large surface area of the sample and a large amount of water available near the surface promote a high evaporation rate, which decreases when the moisture available near the surface is reduced during drying. The type of solvent present, or the composition of the solvents, the solutes in the poured mixture and the heating rate also affect the drying rate. In the papers available for this review, the authors do not report the control of parameters such as humidity or temperature during drying. They do not assess the effect of drying time on film structure and quality. During prolonged drying, intermolecular attractive and convective forces can lead to self-aggregation and inhomogeneity of the resulting product [36].

Membranes produced by solvent casting based only on a single film-forming polymer tend to have inadequate mechanical properties, which limits their use as drug carriers. The formulations described in the literature, prepared as a polymer blend, show enhanced properties, i.e., swelling or elasticity, compared to the pure film-forming polymer. It has been reported that, for example, PVP mixed with cellulose derivatives such as ethyl cellulose and HPC can produce films with increased flexibility and softer, more durable properties [37]. Kida et al. [31] report fabricating matrices based on polyvinyl alcohol (PVA) with cellulose derivatives, i.e., hydroxypropyl methylcellulose (HPMC) or sodium carboxymethyl cellulose (CMC). PVA has excellent membrane-forming properties and has been blended with selected swelling and gelling cellulose derivatives to tailor the mechanical and physicochemical properties of the developed polymer membranes to the application requirements. The smoothness and softness of the resulting film depended on the viscosity and concentration of the polymer in the formulation. It has been observed that too high a viscosity of the cellulose derivative causes collapse and roughness of the surface of the dried film made from PVA. As the concentration of cellulose derivatives in the preparations increased, they became softer and more viscous, leading to difficulties in separating them from the dish. The carriers prepared with polyvinyl alcohol and hydroxypropyl methylcellulose (PVA-HPMC) showed optimal swelling, elastic properties, mechanical properties and disintegration time compared to a pure PVA carrier [31]. Other developed film carriers based on gellan gum and pectin for triamcinolone acetate applied to the oral mucosa showed a high degree of swelling and good mechanical resistance, elasticity and mucoadhesive strength. The films had a high sorption capacity due to hydroxyl and carboxyl groups in the molecules of both polymers. The results indicate an effect of gellan gum on the swelling increase. In addition, the release test has confirmed that the carrier could release triamcinolone acetate in a controlled manner [38]. Table 1 presents examples of polymer matrices for dental topical applications produced by solvent casting.

### 2.2. Freeze-Casting Method

Lyophilization is a technique for preparing dry matrices/materials, i.e., sponge, wafers with a porous structure, expanded specific surfaces and interconnected pore channels or pore gradients, formed by the sublimation of ice grains [43,44,45]. The structure of the carrier prepared by lyophilization corresponds to the sublimation of ice crystals, the growth of which is related to the processing parameters, i.e., the cooling rate of the substrate material and its freezing temperature. The diameter, shape and distribution of the pores and their possible interconnections also depend on the type and concentration of excipients. The choice of water as a freeze-casting liquid is often attributed to its environmental friendliness, low cost and the potential for obtaining a large variety of pore structures [46].

**Figure 2:**
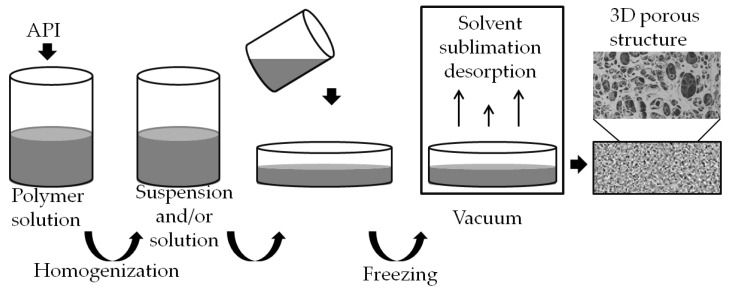
Freeze-casting, lyophilization process (LM).

Lyophilization is a multi-step process involving substrate freezing at atmospheric pressure, pre-drying (sublimation) and drying (desorption) under reduced pressure (vacuum) (Figure 2). Compared to the solvent-casting method, which is carried out under atmospheric pressure at room temperature or higher, the parameters affecting the quality of the final product are more precisely controlled and monitored throughout the freeze-casting process. Maintaining optimum parameters, e.g., temperature and pressure in the chamber during the entire desiccation, results in carriers with desired and reproducible characteristics and high rehydration capacity. In addition, removal of the desired amount of solvent does not require increasing the temperature, eliminating thermal effects that can affect the stability of thermally sensitive substances such as peptides and protein drugs [47].

In the first step of lyophilization, the particle suspension and/or solution containing the dissolved polymer is placed in a mold and cooled to a temperature (value) below the freezing point of the solvent. Cooling the base of the mold promotes solvent solidification along the thermal gradient and unidirectional (anisotropic) crystal growth/solidification, i.e., from the bottom towards the top surface of the sample. When solidification (freezing) begins, the solidification rate is high. As a result, suspended (and/or dissolved) particles are absorbed by the solidification front, resulting in a thickening of the particles in the layer at the base of the sample and an increase in viscosity at the lower surface. The solidification rate then decreases, and particles are pushed (rather than absorbed) through the solidification front, creating an area of particle (and/or molecule) accumulation before/in advance of the front. The resulting crystallites of the active substances and excipients can form eutectic mixtures with the solvent (ice), and each can crystallize as a single component. As solidification progresses, the remaining particles (and/or molecules) concentrate in the intercrystalline space, where the intercrystalline fluid eventually solidifies as well. Further lowering of the temperature leads to supercooling of the condensed liquid and forming an amorphous solid between the crystals (crystallites), the so-called enamel. When the base and sides of the mold are cooled, solidification starts randomly throughout the suspension (mixture). It occurs in random orientations (isotropic), with no preferential crystal growth direction observed [46,47]. During the solidification of the suspension or solution, the structural characteristics of the freeze-cast matrices are established, their walls becoming templated due to the rejection of particles and/or molecules by the solidifying liquid. When the temperature is rapidly reduced during freezing, heat exchange within the product is rapid, and crystal growth is inhomogeneous, reducing the homogeneity of the matrix structure formed. Similarly, when the temperature is slowly lowered, large, regular crystals gradually form, which translates into pore size and an increase in the homogeneity of the resulting matrix. It has been observed that when the freezing temperature is reduced to −80 °C, cracks and fractures always appear in the structure of the resulting matrices [46]. In a subsequent pre-drying step under reduced pressure to a value below solvent evaporation, the heat needed to sublimate the ice inside the product is supplied to the frozen material. It is important that, at this stage, the drying temperature does not exceed the melting point or glass-transition temperature of the resulting crystallites or amorphous forms. This treatment does not reduce the viscosity of the molded matrix and preserves the rigidity of its structure while limiting the formation of internal cracks, damage or kinks. The pores created during drying increase the gas permeability of the subsequent layers, facilitating further elimination of solvent from the product. However, the larger the surface area of the material and the thinner the layer, the more effective the drying is. Once the moisture content of the product is reduced to 7–8%, the material drying begins, for which the drying rate is reduced, and the heat supplied is simultaneously used to raise the temperature of the material and sublimate (ice). The chemically bound water remaining in the product is removed by evaporation or desorption, and the final moisture content of the resulting material ranges from 1.5–3.62% [47,48].

When the solvent is removed, the pores of the cooled materials are spherical throughout, with a structure of single equiaxial cells or connected in a network (mesh) structure. The morphology of the matrices produced by freeze-casting is related to the type and concentration of the selected excipients and the freezing process conditions [18]. In an experiment by Kida et al., gelatin-based matrices with cellulose derivatives were studied. It was observed that higher amounts of sodium carboxymethyl cellulose and hydroxyethyl cellulose (HEC) added to the gelatin resulted in a small number of different-sized holes in the matrix, while the use of higher gelatin concentrations correlated with obtaining matrices with a denser, more homogeneous porous structure with a large number of small holes. Pore size increased with the increasing amounts of CMC and HEC added to the gelatin mixture. Catanzano et al. analyzed sodium alginate and sodium hyaluronate HA sponges loaded with tranexamic acid prepared from a cross-linked hydrogel by freeze-drying. The increase in pore size in the resulting matrices was associated with a higher amount of HA in the formulation of 10–20% [49]. Collins et al. noticed that the change in pore morphology of hyaluronic acid matrices depends on the freezing rate. When the temperature was lowered to 0.6 °C/min, the pores were more homogeneous and smaller than in matrices frozen at 3.4 °C/min [44]. Table 2 provides examples of polymer matrices, i.e., sponges and wafers, for dental topical applications fabricated by freeze-casting.

### 2.3. Electrospinning

**Figure 3:**
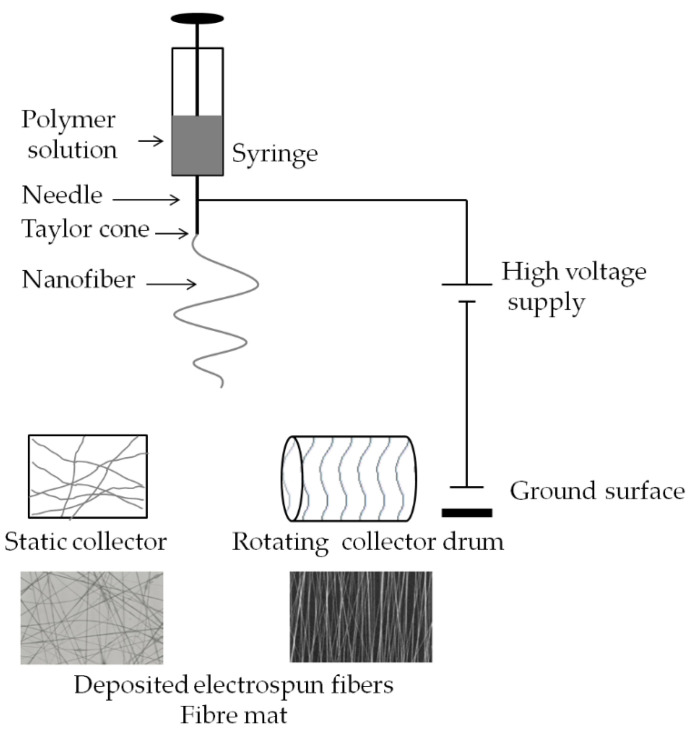
Electrospinning process with various types of collectors: static (**left**) and rotating drum (**right**).

Electrospinning is considered a promising alternative to the solvent-casting method in the manufacture of polymer carriers. This method makes it possible to produce, without using plasticizers, among other things, amorphous fibrous films, membranes, patches with high elasticity and plasticity, and structures with a higher specific surface area compared to those obtained by solvent casting. In the electrospinning method, the aforementioned carriers are created by the controlled spinning of relatively thin nanofibers (typically ~100 nm in diameter), whose layers are arranged in different forms. In electrospinning, the choice of polymer and solvent and the process conditions can affect the mechanical properties of the resulting materials and their behavior after application [54,55]. Solvents used in the method include water, alcohols, acids, ketones, esters, dimethyl sulfoxide, acetonitrile, chloroform, benzene, hexane and diethyl ether, among others. In order to extract nanofibers from a polymer solution or melt, the method uses applied voltage in an electric field. As a result of electrostatic forces, a droplet of solution fed from the device’s needle in an electric field takes the shape of the so-called Taylor cone. The electrically charged jets exiting the Taylor cone are then subjected to stretching and spinning processes, forming a long and continuous strand of polymer threads gathered onto a collector. It should be mentioned that the evaporation of the solvent from the jet, in the case of electrospinning from the solution, accompanies these processes. This process, along with simultaneous stretching, causes a significant reduction in jet diameter and fiber formation. The fiber is attached to a grounded collector and, after some time, a non-woven mat consisting of randomly ordered nanofibers (Figure 3). Despite the simplicity of electrospinning, several parameters can significantly affect fiber formation and structure (see Table 3). These parameters can be divided into three groups: processing, solution and ambient parameters. Solution parameters include the molecular weight and concentration of the polymer, the type and properties of the solvent, viscosity, conductivity and the surface tension of the spinning solution [56,57]. Processing parameters include the applied voltage, the distance from the needle tip to the collector, the needle’s inner diameter, the solution’s flow rate [56] and the type of collector [57]. Ambient parameters include the temperature and humidity of the environment in which electrospinning takes place [56,57,58]. Table 3 lists the factors influencing the properties of fibers manufactured by electrospinning, subdivided into processing, solution and ambient parameters. Each factor can significantly affect fiber morphology. Hence, understanding electrospinning and the appropriate choice of parameters can produce fibers with the desired morphology and diameter.

The value of applied voltage is one of the main parameters of the electrospinning process. The voltage level determines the strength of the electric field between the needle and collector and the velocity of the resulting polymer jet [57]. A suboptimal value of the electric field contributes to defects, e.g., beads in the spun fibers or instability of the formed spinning solution jet [59]. The shape of the initiating droplet changes with the spinning conditions: voltage, viscosity and flow rate [56]. A minimum voltage, the so-called threshold voltage [56] or critical voltage [57]—approximately 6 kV for most polymer solutions—is necessary to balance surface tension and Taylor cone formation. The critical voltage value varies from polymer to polymer. The higher voltage provides stronger repulsion of electrical charges, which is desirable for yielding long fibers with small diameters [57]. Then, at low solution flow rates, the resulting jet is stretched more, the solvent evaporates faster, and fibers with smaller diameters are formed [56]. Lower voltages induce a lower acceleration of the solution jet and a longer electrospinning time. If the jet elongation time is longer, the fiber stretches and elongates more before it settles on the target screen (collector). This promotes the formation of thin fibers. In turn, a weaker electric field increases the diameter of the fibers [57]. The flow rate of the solution also influences fiber size and morphology and affects its porosity and shape [56]. In order to prepare homogeneous nanofibers, the so-called critical flow rate of the polymer solution must be achieved. Its value varies depending on the polymer system used. Increasing the rate above the critical value leads to an increase in pore size and fiber diameter and the formation of beads (this is caused by incomplete drying of the solution jet during the passage between the needle tip and the metal collector). The flow rate of the solution jet, at a given voltage, helps to stabilize the Tylor cone formed [60]. As the flow rate of the solution increases, there is a corresponding increase in the diameter of the fiber, and the size of the beads grows. At high flow rates, the amount of solution flowing out of the end of the capillary increases, causing the jet to be slightly stretched and to take longer to dry due to insufficient time for evaporation. The solvent, which does not have time to evaporate from the spun material, causes the fibers to become entangled, and consequently, webs form instead of fibers [56,57]. Therefore, it is more advantageous for the electrospinning process to maintain a selected optimum flow rate, in which case it takes longer for the solvent to evaporate completely, and the fibers do not become entangled [56,57]. Reducing the inner diameter of the needle also affects the diameter of the fibers. If the diameter of the needle opening is too small, the surface tension of the extruded droplet increases and a higher voltage is required to produce a Taylor cone and initiate the solution jet. As a result, the acceleration of the jet decreases, which increases the time needed to stretch the fiber before it is gathered on the collector. It should also be borne in mind that the extrusion of a drop of solution at the tip of the needle may not be achievable when the diameter of the needle opening is too small [57]. Table 4 and Table 5 indicate the optimized technological parameters of electrospinning and examples of carriers for dental applications produced by this method.

### 2.4. 3D Printing

**Figure 4:**
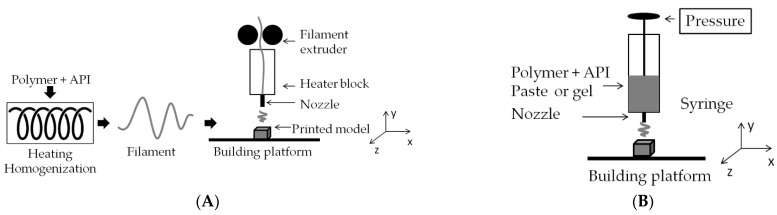
Three-dimensional printing, fused deposition modelling (**A**) and semi-solid extrusion (**B**) method.

Three-dimensional (3D) printing technology makes it possible to produce a customized 3D object based on the chosen material, a specific computer-aided design (CAD) and precise manufacturing. The 3D printing process begins with the design of a 3D model using CAD software. The model is then transformed into cross-sections and sent to a 3D printer, which deposits layer upon layer of the selected material to produce the object. In dental applications, the most widely used methods for printing matrices and films are based on fused deposition modelling (FDM) and semi-solid extrusion (SSE) [68,69,70] (Figure 4).

FDM involves forcing a thermoplastic filament through a printer head heated to a temperature above the melting point of the polymer and moving in three planes: X, Y and Z. The material is then retained on a build platform at a much lower temperature, allowing it to solidify and fuse with subsequent layers until a programmed three-dimensional object is formed. It is necessary to strictly control parameters such as printing speed, single-layer thickness, filling level and the temperature of both the printer head and the build platform [20,71]. The process’s ease of use, the availability of this type of printer, the lack of a need for processing of the final product and the relatively low cost of manufacturing have made it the most popular method in 3D printing technology today. By designing the composition of the filament, it is possible to adjust the dose of the active substance individually for any shape and size [21]. The major limitations associated with FDM use include the need for prior preparation of incorporated filaments using hot-melt extrusion (HME) under controlled pressure and temperature. This poses a risk of thermal degradation of the substance. Hence, a prerequisite for successful HME processing is to select the processing parameters so that the decomposition temperature of the compounds used is not exceeded [72]. The thermoplasticity of the processed material can be improved by adding plasticizers, bearing in mind that their addition also alters the glass-transition temperature of the polymer, changing its elasticity [71]. The HME temperature is usually set at 15–60 °C above the melting temperature (Tm) of semi-crystalline polymers or the glass-transition temperature (Tg) of amorphous polymers. The following stages can be distinguished in the melt extrusion process: (1) feeding the fragmented substance mixture through the hopper, (2) mixing, grinding, particle-size reduction and kneading, (3) flow through the nozzle and homogenization, and (4) extrusion from the nozzle, cooling and further processing [73]. Individually designed processing conditions for the mixture inside the extruder cylinder, including several heating zones as well as different screw configurations, allow the temperature and flow of the material through the extruder to be varied freely, resulting in mixing at the molecular level, which contributes to improving the solubility of poorly water-soluble substances. Before the extrusion process begins, the individual heating zones of the extruder are programmed to set temperatures at which the material being processed is plasticized. As the material moves along the barrel, heat energy is also generated by the shear forces generated by the rotating screws. Efficient mass flow is ensured by the high friction along the inner surface of the barrel and the low friction created between the screws, which can be adjusted by varying the temperature of the individual zones to achieve good flow properties for the mixture. The transport and mixing properties of the screws depend on the angle of displacement between the individual elements, so changes to their configuration are warranted to improve the melting and mass flow through the extruder. This is important because solidified polymer components can block the channel of the extruder, and too high a melt viscosity increases the torque in the extruder, leading to the overloading of the screws and consequently stopping the extrusion process. On the other hand, if the viscosity is too low, it prevents the compact filament from extruding through the nozzle. 

Semi-solid extrusion (SSE) produces an object by extruding a gel or paste-like material using a syringe-based printhead. In the literature, SSE is also known as pressure-assisted microsyringe (PAM) printing, robocasting or robotic material extrusion, cold extrusion-based printing, hydrogel-forming extrusion, soft-material extrusion, melting solidification printing process, direct ink writing, hot-melt ram extrusion, hot-melt pneumatic extrusion and micro-extrusion [71,74]. During extrusion, the material hardens so that further layers are applied during printing [75]. The key differences between SSE and other 3D techniques relate to the semi-solid starting materials used and the low temperature of the printing process [76]. The challenge in this technology is to select the suitable composition of the starting material so that its rheological properties allow it to be processed during printing. The process of extruding material through a nozzle is usually based on pneumatic or mechanical systems. Pneumatic extrusion systems use compressed air and extrusion using a valveless or valve-based configuration. Mechanical systems use a mechanical force exerted directly on the top of the syringe to push material through the nozzle. Mechanical systems are simpler and cheaper, and their use of a piston can also provide greater control over critical extrusion parameters, such as the force exerted on the top of the syringe generating the resulting material flow. The pressure magnitude allows control of the pressure desired to achieve the flow rate, after which the pressure must be kept constant and without large oscillations to ensure the required quality of the final product. An increase in applied pressure observed in the process may indicate nozzle clogging. In contrast, a sudden pressure drop may indicate the presence of air inside the syringe or nozzle, reducing the amount of mass deposited. Clogging or intermittent material deposition is undesirable and leads to inconsistent or failed prints. Therefore, pressure control streamlines the printing process by establishing correlations between the applied pressure and the quality attributes of the object, such as weight, dimension and dose. Both material properties (e.g., viscosity) and processing parameters (e.g., the nozzle of defined length and tip diameter, flow or extrusion speed and extrusion temperature) can affect the printability of materials. Low-viscosity materials can be easily extruded, but the final objects may deform. On the other hand, if the viscosity is high, the material may not flow. Applying a significant mechanical force to the extrusion can improve material flow. Similarly, heating the raw material can also facilitate material flow if its viscosity is temperature-dependent. Examples of carriers produced by 3D printing are provided in Table 6.

## 3. Assessment Methods for Polymer-Based Dental Carriers

The dry carriers produced by the methods described above were subjected to evaluation, depending on the carrier form, according to, among other aspects: (1) their physicochemical properties, i.e., morphology, formulation composition and component distribution, pore size and volume, mechanical properties, swelling and disintegration, mucoadhesion, and excipient–drug interaction; (2) their pharmaceutical properties, i.e., drug load uniformity, drug release and stability; (3) their biological properties, i.e., antibacterial and antifungal activity of formulations, and cytotoxicity; and (4) in vivo assessment of polymer-based matrices, i.e., animal experimental evaluation and clinical study. It should be stressed that the choice of a method for testing the properties of a carrier should allow for a comprehensive evaluation of the carrier in order to achieve the desired characteristics of the designed formulation for dental applications, taking into account the dynamic conditions at the application site in the oral cavity.

### 3.1. Physicochemical Properties of Matrices 

#### 3.1.1. Morphology of the Surface of Fabricated Matrices and Component Distribution

Direct non-invasive optical methods are used to analyze the surface morphology of the matrices. An optical microscope, with the appropriate magnification and contrast settings, makes it possible to confirm the smoothness of the observed area of the sample, observing creases and roughness on it. At 8–40 times magnification and 200 nm resolution, changes/objects larger than 10 μm in diameter can be observed on the sample surface under an optical microscope [81]. 

Scanning electron microscopy (SEM), with a magnification range of 10–100,000×, is used to observe the surface of carriers and identify changes ranging from nanometers to millimeters in size, to assess pore morphology and for preliminary analysis of pore size. In SEM, a focused beam of primary electrons is directed at the surface of the test sample, scanning the surface layer of the material. An accurate picture of the surface structure is obtained based on the analysis of the signals and secondary and backscattered electrons. In order to increase the electrical conductivity, the carrier is coated with a thin conductive layer, e.g., gold, before testing, and the analysis is carried out in a vacuum. The sputtered layers, which have a thickness range of 0.01–1 nm, not only act as an electrical conductor but also protect the test sample from the thermal effects of the electron beam. The SEM image provides information about the porosity and cracks of the analyzed area, and the homogeneous or heterogeneous distribution of pores in the material and their shape [82]. Transmission electron microscopy (TEM) is also used to analyze the morphology of fine structures and membrane layers, in which an electron beam passes through the sample, providing images of deeper sample structures—up to 1 μm below the surface, with a resolution of <1 nm. The disadvantage of this method is the small sampling volume (ca. 2–5 μm in the three dimensions), which does not give an overview of the whole membrane structure [83]. 

**Figure 5:**
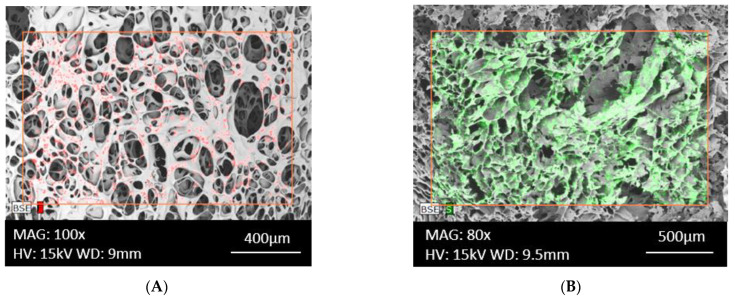
SEM/EDX mapping of two porous matrices from polymers: pullulan, sodium alginate and methylcellulose with triamcinolone acetonide (**A**) and methylene blue (**B**). Elemental mapping of the chemical composition was carried out for the fluorine atom in the matrix with the triamcinolone molecule and the sulfur atom for the matrix with methylene blue.

Electron microscopy combined with X-ray microanalysis (SEM/EDX), which uses an energy-dispersive X-ray spectrometer as a detector, is also used to evaluate carriers. This method makes it possible to record characteristic X-rays from the surface of the sample and to determine the chemical composition of the carrier surface, the homogeneity of distribution and the distribution map of elements in the area under study with micrometer spatial resolution. Qualitative microanalysis produces a clear X-ray spectrum from a selected sample section and provides a map of elemental distribution, including imaging of the distribution of the constituent active substances in the material under investigation. In quantitative analysis, the elemental concentration is calculated from the proportional dependence of the intensity of the characteristic X-rays on the elemental content of the volume analyzed. After careful calibration with appropriate standards, the microanalyzer software performs conversions of peak intensities to elemental percentages [84]. EDX chemical composition analysis allows the detection of elements at around 0.1 wt% and provides quantitative results using the aforementioned calibration [85]. Figure 5 shows an example of SEM/EDX imaging of two porous matrices prepared from the polymers pullulan, sodium alginate and methylcellulose, with the active substances triamcinolone acetonide and methylene blue, respectively. Imaging of the distribution of the API components in the matrices was performed based on elemental mapping of the chemical composition of the active substances, in this case for the fluorine atom and the sulfur atom.

#### 3.1.2. Pore Size and Volume Assessment

Surface porosity, heterogeneity and roughness are significant aspects in studying materials and correspond to the volume of void space, which may contain fluid or air, relative to the total volume of the material. The morphology of the porous structure influences its water-related properties, such as the degree of swelling and matrix leaching. The larger pore volume and porous structure facilitate water uptake into the matrices [51]. Pore size and homogeneity affect the biological activity of the produced matrices. For example, matrices with homogeneous porosity are more rapidly absorbed and biodegraded than carriers with less homogeneous structures [51]. The porous structure of the material influences a number of matrix properties, such as sorption capacity and mechanical properties. The pore system can be classified due to its accessibility to the environment into closed and open and the following geometrical shapes: cylindrical, bottle-shaped, slit-shaped and conical [81]. The process of adsorption of gas molecules (adsorbate) onto the exposed surface of a dry material (adsorbent) is used to study the estimation of pore size distribution. A gaseous adsorbate, i.e., argon, krypton, helium or polar liquids such as water vapor, is used to penetrate the pores and, depending on the type of gas, adsorption allows the pore size to be assessed starting from ca. 0.35–100 nm. In practice, nitrogen at a constant temperature of 77 K as the boiling point of the gas is most commonly used to describe the surface of mesopores with a diameter of 2–50 nm and macropores with a diameter of less than 100 nm. When gradually increasing the partial pressure in the predicted range value, the amount of adsorbed gas is measured, and an adsorption isotherm is plotted. Likewise, when decreasing the pressure, the amount of gas removed is measured, and a desorption isotherm is obtained. Based on the defined adsorption isotherm, the amount of gas adsorbed in the monolayer region of the sample is calculated and, based on equations relating to the pressure at which condensation or evaporation of the adsorbate occurs on the surface of the adsorbent, the pore size distribution of the matrix is estimated. The Brunauer–Emmett–Teller (BET) method is used to measure porosity surface area and calculate the mean pore radius, pore size distribution and total pore volume from the adsorption isotherm. The BET Equation (1) assumes a cylindrical pore shape, and the amount of adsorbed liquid in the gaseous state as a volume is expressed as

Vs/S = r/2 (1)

where S is the specific surface area, Vs is the volume of condensed liquid calculated from the amount adsorbed near saturated vapor pressure, P/Po is relative vapor pressure near 0.95, and r is the mean pore radius. Due to the difficulty in defining the surface area when the pores have different dimensions, some researchers also recommend combining different techniques to improve the accuracy of the porosity description. Gas adsorption techniques indirectly determine pore surface properties and pore size distribution. The limitations of using the gas adsorption method are the inability to measure closed porosity and the need to dry and clean the sample surface of other gases before analysis. Pre-treatment of samples with an appropriate combination of low vacuum and temperature should be used to avoid measurement errors. Outgassing at too high a temperature or under ultra-high vacuum conditions can lead to changes in surface composition, such as the decomposition of components and the formation of surface defects or irreversible texture changes. During the interaction of the gas with the surface of the solid, there may be a change in the concentration of the component at the interface; the adsorbate molecules may give specific adverse interactions in small micropores, surface functional groups and exposed ions, causing erroneous results [81,86,87].

#### 3.1.3. Assessment of Hydrophobic/Hydrophilic Properties 

**Figure 6:**
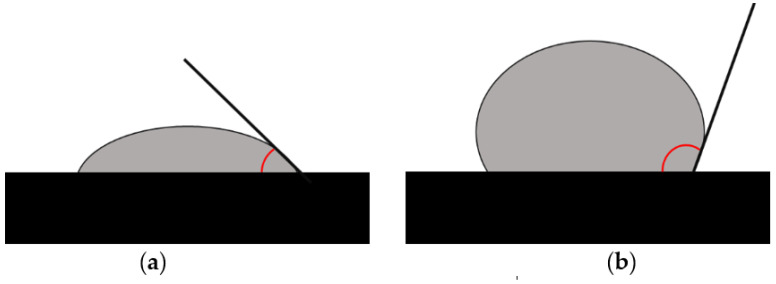
Contact angle when a drop of water is applied to a solid material surface; <90° indicates wetting (**a**), ≥90° indicates no wetting (**b**).

A valuable method for describing carrier surface properties is measuring the contact angle. The measurement data can provide information on the hydrophobicity and morphology of the surface layers of the examined material. The contact angle is measured by determining the tangential angle of the liquid droplet to the solid surface at the base. The value of the contact angle can be interpreted directly using Young’s equation:

*Γlvcosθy* = *γsv* − *γsl* (2)

where *γlv*, *γsv* and *γsl* represent the liquid–vapor, solid–vapor and solid–liquid interfacial tensions, respectively, and *θ_Y_* is the contact angle if the assumptions are met for the surface of the tested carrier, which should be rigid, undeformable, smooth, stationary and unable to change orientation in contact with the liquid. In addition, the surface to be tested and the liquid must not interact, i.e., show swelling or cause extraction of solid components. Importantly, as most of the assumptions for ideal contact angle measurements are violated by the actual measurement conditions, the tests must include the influence of these non-ideal conditions in the analyses. A digital goniometer connected to an image analyzer, which photographs the surface (interface) of the liquid and automatically calculates the angle, is usually used to measure the contact angle of the carriers [88]. In the experiment, samples of test liquids with different surface tensions, e.g., water, glycerol, etc., are applied as droplets to the solid surface of the test material. Then, the contact angle is assessed for the deposited droplets, with a contact angle *θ* of >90° for highly non-wettable surfaces and ∼0° for highly wettable surfaces (Figure 6). 

#### 3.1.4. Testing of Mechanical Properties

The mechanical properties of the matrices, including percentage elongation and maximum force applied at sample break, are determined using a texture analyzer at ambient temperature. In the apparatus, the test sample is held by two clamps placed at a defined distance (ILB) and stretched at a constant speed until it breaks. The strength and elongation of the material before breaking apart (IL) are measured. The maximum elongation (E%) at the break of the matrices was determined using the following equation [51]:

E% = (ILB/IL) × 100 (3)

Young’s modulus (E), also referred to as the elastic modulus, describes the influence of the strain and its force at that strain on the matrix surface and is calculated using the following equation:

E = F × l0/S × ∆l (4)

where F is the applied stress, S is the cross-section area of the film, and ∆l is the amount by which the length of the material changes. Young’s modulus indicates the elasticity of the material. It is the hypothetical strain when the sample is elongated twice, and the cross-sectional area of the sample is assumed not to change. The high value of Young’s modulus (E) indicates the low deformability of the analyzed sample during stretching and compression and describes the polymer carrier obtained.

The matrices are also assessed for flexural strength (folding endurance). For this purpose, the number of folds the film can withstand before it breaks when subjected to a constant load is determined [41]. The flexural strength of the carrier is assessed by a three-point bending test conducted using a texture analyzer. During the test, the apparatus probe moves downwards at a constant speed, bending a long sample placed on two supports. During the test, the force applied and the distance travelled by the probe are recorded. The carrier strength is expressed by the sample stress (FS) during the test. It is calculated using the formula FS = 3 Fg/2wd^2^ [Pa] for rectangular cross-section samples and FS = 8 Fg/πd^3^ [Pa] for circular cross-section samples, where F is the load applied by the texture analyzer, g is the distance between the support points, w is the width of the sample and d is the thickness of the sample.

#### 3.1.5. Matrix Swelling and Leaching/Disintegration Assessment 

Swelling capacity is one of the most important features of polymeric drug delivery systems, as it significantly impacts the release kinetics of the drug incorporated into the carriers. In addition, it is directly related to mucoadhesiveness, as the bioadhesive sites in the polymer chain are exposed during the swelling process when the polymers absorb the carrier. The mechanism of polymer swelling can be divided into three main stages. Initially, water is adsorbed onto the surface of the carrier; then, as a result of surface saturation, the liquid migrates into the matrix, interacting with the polar groups of the polymers, which weakens the intermolecular hydrogen bonds in the polymer chains and leads to their relaxation. In the third stage, the spaces created between the distant chains facilitate the penetration of water or other applied medium into the deeper layers of the matrix [38].

The swelling ratio—understood as the increase in weight of the sample at time points after soaking—is determined by placing an accurately weighed sample (mounted on the tip of a needle) in a specified volume of water to soak at 37 °C. The weight of the sample is recorded once or at intervals of 1–6 h after immersion in the liquid and then every 24 h thereafter until equilibrium is reached. To measure weight over time, the removed samples are drained of excess water using tissue paper and re-immersed in water after weighing. The percentage swelling ratio (SR %) at each time point is calculated using the following equation, where W is the mass of the swollen sample and W0 is the mass of the initial dry sample [38,39,41,49,51].

SR% = [(W − W0)/W0] × 100 (5)

The leaching/disintegration time resistance of polymeric drug carriers intended for topical mucosal administration should also be considered in evaluating the carriers. Due to the effects of saliva, tongue movements and swallowing, it is desirable to keep the applied carrier and the drug in contact with its site of action longer. Carrier disintegration is the time it takes for the carrier to dissolve fully. Samples of precise size (mm) are immersed in (mL) saline buffer or artificial saliva volumes and incubated at 37 °C in a sealed dish with horizontal shaking. The test continues until the original carrier dissolves entirely in the medium [51].

#### 3.1.6. Mucoadhesion Evaluation

Testing of the mucoadhesive properties of the carriers employs a texture analyzer and mucous membranes of animal origin, human gastric mucosa, synthetic membranes including cellulose, disks of compressed mucin and hydrogels. In order to test adhesion, the detachment force of the investigated carrier from the applied membrane surface is recorded in multiple repetitions, and the surface area is calculated under the force-distance graph. The experimentally obtained surface area is directly proportional to the force required to break the bonds formed between the applied film surface and the carrier (detachment force evaluation) and allows comparison of the adhesive properties of the analyzed formulations [89,90].

Several other methods have also been published for testing the mucoadhesion of polymer carriers using, among other things, a modified surface tensiometer, a cone/plate viscometer, an atomic force microscope (AFM) and based on the evaluation of the leaching rate of the carrier deposited on the test film [90,91]. However, a texture analyzer appears to be the most versatile method, despite the high coefficient of variation of the results, often amounting to around 60%, which implies a significant variation in the characteristic being analyzed and the need for numerous repetitions.

#### 3.1.7. Methods for Assessing Drug–Excipient Interactions and the Stability and Physical State of Drugs in the Formulation

Potential excipient–drug interactions in the resulting carriers are investigated using Fourier-transform infrared spectroscopy (FTIR). FTIR spectra at room temperature are recorded in the range of 4000–400 cm^−1^ for pure APIs, excipients, i.e., polymers, formulation components and samples of the tested formulation of films, matrices and sponges [31,38,42,49]. Characteristic peaks of the API and all excipients are observed on the infrared spectrum of the test material, with the absence of the appearance of new bands or shifts in the characteristic peaks in the samples indicating that the components of the layers are not reacting with each other to form new chemical compounds. Therefore, it can be speculated that drugs and polymers are compatible and can be formulated in the examined carrier. On the other hand, observed changes in the spectrum of the materials are the result of bonds formed between the components. Kan et al. noted changes in the spectrum of glutaraldehyde cross-linked chitosan films, indicating that the ingredients used to fabricate the films were compatible, given the successful cross-linking. In this case, an imine bond was formed between the amine groups of CS and the aldehyde groups of the glutaraldehyde condensation product. In contrast, an unsaturated α-β double bond (−C=C−) was formed by dehydration and condensation of the glutaraldehyde [42].

The compatibility, degradation, stability and physical state of drugs in the formulation are determined by differential scanning calorimetry (DSC) [42]. The basic principle of DSC is that a sample is exposed to a thermal signal, and the response is measured in terms of the energy and temperature of thermal events that occur over the temperature range or time interval under study. DSC is one of the primary tools used to describe the state of the matrix, with polymorphism and drug incorporation into materials. DSC enables the analysis of potential polymorphs and the amorphous form of the preparation’s ingredients and glass transition of the film. Shifts in the endothermic peak and peak area broadening directly indicate the phase transition as well as the molecular interaction of the drug molecule trapped inside the formulations. DSC reveals whether the components are in an amorphous form inside the material. The absence of an endothermic peak of the substance in the DSC heating profiles studied, where the raw powder has its melting peak, indicates that the relevant active pharmaceutical ingredient has an amorphous form. In DSC, the shift in the endothermic peak observed after the drug is introduced into carrier formulations may indicate its increased thermal stability.

A key parameter for quality control of the resulting polymer formulations is also the amount of residual moisture, which can correlate with poor product stability. Thermogravimetric analysis (TGA) can be employed to estimate the amount of residual moisture in lyophilized matrices. The operation of thermogravimetric analysis instruments involves recording the change in mass of the sample with a change in temperature or time. The test provides information on the content of volatile materials, such as residual moisture in the material. The high residual water content of the materials can act as a trigger to initiate and accelerate crystallization, which can cause polymorphism within the system, resulting in product instability. Water is also an effective plasticizer that significantly lowers the glass transition temperature (Tg) of the active compound and excipients by increasing molecular mobility, resulting in product instability, including the possibility of reverse melting during the primary drying step during lyophilization [92].

Proton techniques (^1^H NMR) and solid-state NMR techniques (^13^C CP/MAS NM) can be used to study drug stability during the film-casting process [89]. Comparison of the relative intensities and chemical shifts of the ^1^H spectra for solutions of components alone and the mixture of components can suggest whether the chemical structure of the drug was unaffected during the film-casting process. NMR spectral analysis of the drug extracted from polymer films also makes it possible to assess whether the studied drug was unaffected during its release from the carrier. In the solid-state spectra of the pure drug and mixtures of components, the relationship between the spectra and the assessment of the physical state of the drug in the carriers is analyzed. The similarity of the lines (both shape and position) in the drug powder and the mixture suggests that the drug is dispersed in the film as small crystallites rather than in a true solid solution [93].

### 3.2. Pharmaceutical Properties

#### 3.2.1. Drug Load Uniformity

Achieving a homogeneous distribution of the API in the matrix can be difficult, as the drug crystals in the matrix structure may be suspended or completely dissolved in the material. Pharmacopeial uniformity testing of dosage units is suitable for validating dose uniformity in batches of manufactured matrices. The uniformity of drug content, between batches and within batches, is determined by dissolving individually weighted material samples of a defined area in a suitable solvent, often water, in a volumetric flask. The resulting solutions are filtered, the filtrate is diluted accordingly, and the API content is determined according to a validated substance determination test [41,51]. The mean and standard deviations are calculated within the assumed value range for the tested series of samples.

#### 3.2.2. Study of Drug Release/Pharmaceutical Availability of API in Developed Carriers

The in vitro drug release studies were performed using an FPXI apparatus type 1 [94] in multiples for each formulation. The matrices were immersed in water, phosphate buffer (pH 6.8) and artificial saliva solution maintained at 37 °C ± 0.5 °C, with a stirring speed of 50–200 rpm. During the test, the sink condition was maintained throughout the experiment and samples were taken over time. After filtration, the drug content of each sample was analyzed using ultraviolet-visible spectrophotometry and HPLC or different methods. Release from the carriers can also be carried out using Franz cells through a synthetic or natural film at a temperature of 37 °C ± 0.5 °C, with a stirring speed of 100–600 rpm [39]. Samples taken at the time points are determined by the chosen method, similar to the type 1 apparatus. The mathematical approaches for assessing drug release from manufactured matrices include the first-order kinetic model lnQ = lnQ_0_ − kt and the zero-order kinetic model Q = Q_0_ − kt, where Q is the amount of drug released, k is the release constant, and t is time. The data are then fitted to the Higuchi equation Q = k√t, where Q is the amount of drug released, k is the release constant, and t is time, and to the Korsmeyer–Peppas equation (power law) Q = kt^n^, where Q is the fraction of drug released at time t, k is the structural and geometric constant, and n is the release exponent [51]. The parameters derived by fitting the experimental data from the release study to different kinetic models play a significant role in understanding the process mechanism. The parameter values of selected mathematical models are used to compare the release profiles of substances from carriers, and the data obtained, such as the release exponent n in the Korsmeyer–Peppas model, allow assessing whether the drug will be released by erosion, diffusion or indicates a greater contribution of one of these processes. Table 7 shows the n value of the mechanism of drug release from the polymer film in the Korsmeyer–Peppas model.

#### 3.2.3. Stability Study

Material stability studies are often performed under the storage conditions of the medicinal product in selected packages (see Table 8) [92,93]. At each interval, the carriers are observed for changes in parameters such as physical appearance, physicochemical properties and drug content. Changes in these parameters make it possible to assess the influence of the storage conditions selected in the stability test of material properties, i.e., elasticity, mechanical strength, deformation, moisture, formation of polymorphs or changes in substance content over time. In practice, this helps select storage conditions and the type of packaging to ensure that the required formulation quality is preserved.

### 3.3. Biological Properties

#### 3.3.1. Evaluation of the Antibacterial/Antifungal Activity of the Formulations

The antimicrobial activity of the preparations is investigated using the agar diffusion test against *Staphylococcus aureus* (Gram-positive bacterium) and *Escherichia coli* (Gram-negative bacterium), which are commonly found in the oral flora, and antifungal activity using *Candida albicans* (*Saccharomycetes*), which is found in the digestive tract. First, the Mueller–Hinton agar is poured onto sterilized Petri dishes, and the strains are inoculated onto agar plates. The weighted test preparation is placed on agar plates under aseptic conditions and incubated at 37 °C for 24 h under aerobic and anaerobic conditions. The diameters of the zones of inhibition are measured at the end of the incubation using digital calipers, and the zones of inhibition of bacterial/yeast growth for the drug- and placebo-incorporated matrices are compared and correlated [19,31,51]. An antifungal assay can be performed using *Candida albicans* cultures prepared on Sabouraud dextrose agar plates. After being dissolved in dimethyl sulfoxide (DMSO) and then diluted in RPMI medium, the prepared samples are transferred in duplicate to the wells of a 96-well microplate. *C. albicans* inoculum is added to the resulting samples, and the test plates are read at 630 nm before and after incubation for 48 h at 37 °C [98]. The growth percentage is calculated and plotted against the concentration tested to obtain the half-maximal inhibitory concentration (IC 50) and minimum inhibitory concentration (MIC).

#### 3.3.2. Cytotoxicity Testing

A series of international standards for medical devices (ISO 10993) have been published by the International Standards Organization (ISO). Three types of cytotoxicity tests are stated in ISO 10993-5: extract, direct contact and indirect contact tests (including agar overlay assay and filter diffusion). The choice of one or more of these categories depends upon the nature of the sample to be evaluated, the potential site of use and the nature of the use. In vitro techniques used to assess cytotoxicity are summarized in Table 9.

The potential cytotoxic effects of the developed formulations can be tested using selected reference cell cultures of gingival fibroblasts and osteoblasts (L929 and U2-OS). In the test, a carrier with the active substance is introduced into the cell culture, and after incubation, the cells are stained with neutral red, for example. The dye penetrates only the living, metabolically active cells. The culture is then terminated by treating with a mixture of acetic acid and ethanol, and the amount of dye present in the living cells is measured using colorimetry. At the same time, a culture containing a placebo carrier sample is run under analogous test conditions. The cytotoxic activity of the evaluated formulations is determined in relation to a control culture not exposed to the tested carrier [51,99,100].

**Table 9:** In vitro techniques used to assess cytotoxicity [101,102,103,104].

Test	Test Category	Assay/Detection Method
NR Neutral red	colorimetric	- Based on red uptake by lysosomes of the living cells; - The NR are extracted from lysosomes for quantitative measurement of cell viability.
LDH Dehydrogenase lactate	colorimetric	- Measurement of the activity of cytoplasmic enzyme; - Dehydrogenase lactate leak into the culture medium due to changes in cell membrane integrity (membrane permeability) and cell lysis and converts the terazol salts in the medium into colored formazan; - The color intensity of the formazan is an indicator of cell viability.
NAG *N-Acetyl-*β-*D*-Glucosaminidase	colorimetric	- Demonstration of the lysosomal enzyme N-acetyl-beta-D-glucosaminidase presence in a culture medium, which is possible when the cells are damaged, indicates the lack of cell membrane integrity.
SRB *Sulforhodamine B*	colorimetric	- Stoichiometric binding of SRB dye to proteins; - The protein dye sulforhodamine B binds electrostatically and pH dependent on protein basic amino acid residues of trichloroacetic acid-fixed cells, the amount of dye extracted is a proxy for cell mass and thus the number of cells in a sample.
ATP Adenosine triphosphate	bioluminescent	- Luciferase/luciferin reaction used to measure the amount of *ATP* in living cells; - The intensity of the light emitted during the oxidation of luciferin with ATP is proportional to the amount of ATP in living cells.
MTT/MTS	colorimetric	- Measurement of the activity of mitochondrial enzyme; - Succinate dehydrogenase in the living cells reduced colored tetrazole salt to insoluble (MTT) or soluble (MTS) in water formazan form; - The color intensity of the formazan is an indicator of cell viability.

## 4. In Vivo Assessment of Polymer-Based Matrices 

### 4.1. Evaluation of Therapeutic Effects of the Examined Material In Vivo

Takashima et al. evaluated the chemopreventive potential of an apigenin-loaded film fabricated by 3D printing in animal experiments. To this end, the researchers first induced the formation of an oral carcinogenesis model on the tongues of rats after administering, among other things, a solution of 4-nitroquinoline 1-oxide (4NQO) daily for eight weeks. The animals were then separated into two groups: Group 1 received 4NQO solution for eight weeks and no treatment (n = 6); Group 2 received 4NQO treatment for eight weeks and then had a film carrier applied (n = 6). All rats were euthanized at week 22 after film application and histopathological evaluation of the tongue sections by light microscopy and immunohistochemical evaluation was performed to determine the effects of the material on the expression of the tumor and inflammatory markers Ki-67 (MIB-1), nuclear factor kappa B (NF-κB) and 8-hydroxy-2′-deoxyguanosine (8-OHdG) [80]. Assessing the presence of Ki67 protein on tumor cells is helpful in monitoring treatment efficacy and the risk of disease progression. Higher labelled amounts of Ki67 protein are associated with an increased risk of progression. Increased expression and activation of NF-κB is also a hallmark of progressive inflammation associated with tumor progression. In addition, 8-OHdG is one of the most common markers for assessing the extent of oxidative DNA damage, and its increase indicates disease progression.

Zhang et al. described the evaluation of the therapeutic effect of the material, which was a bilayer mucoadhesive buccal film containing a combination of ornidazole (OD) and dexamethasone sodium phosphate (DEX-P) in rabbit studies. After approval from the Animal Ethics Committee, researchers induced oral ulcer-like lesions in animals by administering 50% glacial acetic acid. They started evaluating the therapeutic effect of the carrier after the initiation of ulceration, dividing the animals into groups: Group 1 with untreated animals as a control group, Group 2 with animals treated with placebo material, and Group 3 with animals treated with tested material with active substance(s). All animal groups, both those to which carriers were applied to the ulceration sites and the control group, were observed over time. The ulceration area was measured, including establishing the mean ulceration area. On day 12 of the study, the animals were euthanized, and biological material samples were subjected to histological evaluation. The therapeutic effect was assessed by the density of neutrophils in the oral sub-epithelial tissue [39].

### 4.2. Clinical Studies

The symptoms of clinical oral lesions vary widely. It is generally necessary to relate the clinical and histopathological picture in diagnosing oral mucosal lesions. Multiple methods are available to assess the evolution of mucosal lesions influenced by treatment. They are based on the evaluation of changes in patients’ subjective assessment, objective medical assessments according to criteria of clinical indicators and comparative image analyses based on standardized dental photography. The most commonly assessed subjective parameter is the change in pain intensity following treatment. For this purpose, the visual analogue scale (VAS) or the numeric rating scale (NRS-11) are most commonly used [105]. In the VAS assessment, the patient marks the rightmost point on a 100 mm horizontal line corresponding to the pain intensity between left ‘no pain’ and right ‘worst pain imaginable’. In the NRS-11 assessment, the patient assigns an integer number from 0 (no pain) to 10 (pain as bad as it could possibly be) to the pain intensity. The patient’s subjective evaluation is recorded in determining the oral health-related quality of life (OHRQOL). This allows linking oral health and the patient’s subjective well-being assessment and makes it possible to assess potential limitations in the social sphere. Psychometric quality of life testing involves the patient completing a questionnaire that has been previously analyzed and validated. It is possible to make an overall assessment with OHRQOL through such multiple indicators and indexes as the Oral Impact on Daily Performance (OIDP), oral health index (OHX), Geriatric Oral Health Assessment Index (GOHAI) and Liverpool Oral Rehabilitation Questionnaire (LORQ). The second group is made up of indicators estimating the impact of specific conditions affecting self-assessment of oral health, such as the Xerostomia-Related Quality of Life Scale (XeQoLS) or the University of Washington Quality of Life Questionnaire (UW-QOL) related to head and neck cancer. In addition, there are health-related quality of life (HRQoL) indicators assessing the physical, psychological and social impact of health conditions on individual well-being, e.g., the European Quality of Life Instrument (EuroQol), 36-Item Short Form Survey (SF-36), World Health Organization Quality of Life (WHOQOL) and Sickness Impact Profile [106]. One of the most popular questionnaires for the overall assessment of OHRQoL is the OHIP-49 with 49 items [107] and its shortened version, OHIP-14 [108].

The Autoimmune Bullous Skin Disorder Intensity Score (ABSIS 1 for assessing the extent of mucocutaneous lesions on a scale of 0 to 150 and ABSIS 2 for assessing the extent of oral lesions on a scale of 0 to 11) is sometimes used in the clinical assessment of changes in the mucocutaneous lesions [109]. Using a periodontometer or caliper, the area of pathological lesions on the oral mucosa is also assessed to some extent by measuring their greatest width and height. The detection of mucosal abnormalities and their true extent can be improved by fluorescence examination with the Velscope Vx system. In the clinical evaluation of the healing of erosive–ulcerative lesions, the efficacy index (EI) is useful, in which the reduction in the size of such a lesion at the follow-up visit is expressed as a percentage, with an interpretation: 100% denotes healed lesion, 99–70%—marked improvement, 69–30%—moderate improvement, and below 30% no improvement [110]. The Mucosal Scarring Index (MSI) has been proposed to assess oral scarring after surgical treatment [111]. Five clinical parameters of the scarring are then assessed on a scale of 0 to 10: width, height/contour, color, visibility of suture marks and overall appearance. In addition to these non-characteristic indices used to assess the treatment of many oral pathologies, parameters specific only to one diagnosis have also been developed, e.g., Thongprasom sign scoring for lichen planus, the lichen treatment efficacy scale by Carrozzo and Gandolfo, the WHO five-point scale for mucositis after chemotherapy and the Newton classification of denture stomatitis. In contrast, the five-year survival rate most simply expresses the effectiveness of holistic treatment for oral squamous cell carcinoma. 

Photometric techniques are currently being developed to describe the healing of oral mucosal lesions more precisely. Digital images of clinical lesions with L-shaped graph paper benchmarks with 1 mm intervals placed around them are transferred into a graphical computer program, and after delineating the lesion edge and filling this outline with a colored marker, it is possible to calculate the exact area of the lesion in mm^2^ [112]. This makes it possible to determine the percentage unhealed index (PUI), expressing the percentage of the lesion not healed at follow-up visits in relation to the baseline. The changes observed following treatment in the fractal dimension and texture (microcontrast) values of mucosal lesions on intraoral radiographs assessed under different light can be used to analyze the comparative effectiveness of treatment methods [113]. 

## 5. Conclusions

This review focuses primarily on the characteristics of the technologies being developed for the production of modern dental carriers, i.e., the solvent-casting method (SCM), lyophilization method (LM), electrospinning (ES) and 3D printing (3DP), and it identifies formulation evaluation methods depending on the formulation type. The location of the application site in the oral cavity varies and includes, among others, the cheeks, the tongue surface, the palate, the alveolar processes of the maxilla and mandible (gums), the floor of the mouth and the periodontal pockets. This area is constantly moistened and flushed with saliva, which exposes the carrier to leaching and poor penetration of the drug into the tissue, and the site dynamics limit adhesion of the formulation and promote rapid removal or accidental ingestion of the carrier. Polymer-based carriers for dental applications are expected to have properties beneficial to oral health also after processing. Hence, formulation development requires a multidirectional analysis of its properties, including (1) physicochemical, (2) pharmaceutical and (3) biological properties and (4) in vivo evaluation, e.g., in an animal model or, ultimately, in clinical application. A comprehensive assessment facilitates optimizing the formulation technology and parameter selection to achieve prolonged retention time of the carrier in the oral environment. Thoughtful and planned assessment of in vitro physicochemical and pharmaceutical properties is essential to clarify observations in clinical trials, enabling consequent selection of the optimal formulation. The development challenge for obtaining dental carriers by the aforementioned methods is still the optimization of the technology for their preparation in order to guarantee:

- Homogeneity of their structure;
- API content of the carrier;
- API stability, including temperature-sensitive substances;
- High drug loading;
- Elimination, for example, of residual solvents.

Advantages, disadvantages, challenges and of potential solutions associated with described methods preparing polymeric carriers summarized in Table 10. 

The technology is also expected to ensure the reproducibility of the process and reduce its multi-step nature, or the need for post-processing of the carrier. The challenge is the development of new approaches exploiting the use of greener and biocompatible solvents such as water, avoiding the use of organic solvents, and using alternative methods, e.g., melt electrospinning without organic solvent, or a low-temperature extrusion method. For technologists working on drug formulation, it is also important to be able to obtain carriers using methods that allow full control of process parameters, including the reduction of process steps, which makes it possible to increase the scale of production and transfer the technology to industrial development.

## Figures and Tables

**Table 1:** Polymer matrices for dental topical applications produced by solvent casting.

Type of Carrier	Polymers/ Excipients	Processing Parameters	API	Ref.
Film	PC, GG	Solvent/temperature: water/50 °C Stirring speed/time: 150 rpm/12 h Drying time/temperature: 24 h/40 °C	Triamcinolone acetonide	[38]
Film	PVA, HPMC	Drying time: 24 h Temperature: 40 °C	Povidone-iodine	[31]
Bilayer film	EC, HPMC, PVA, CS	Anhydrous solvent: ethanol	Ornidazole and dexamethasone sodium phosphate	[39]
Fast-dissolving orodispersible films	DX, MT, HMPC-AS, HPC, Microcrystalline cellulose (Avicel)/GLY	Solvent: methanol Time drying under vacuum: 4–5 h	Amphotericin B	[40]
Multilayer film	CS, PC	Solvent: water Drying time: 25h Temperature: 30–35 °C	Clotrimazole	[41]
Film	CS/propylene glycol	Solvent: 0.5% acetic acid solution Drying time/temperature: 50 °C/24 h	Metronidzole/ levofloxacin	[42]
Monolayer and bilayer mucoadhesive film	CS, HPMC/acetic acid solution glycerin	Monolayer film solvent casting Bilayer films were prepared using double casting	Cefuroxime axetil	[19]

CS—chitosan, DX—dextran, EC—ethyl cellulose, GG—gellan gum, GLY—glycerol, HPC—hydroxypropyl cellulose, HPMC—hydroxypropyl methylcellulose, HPMC-AS—hydroxypropyl methylcellulose acetate succinate, MT—maltodextrin, PC—pectin, PLGA—poly(D,L-lactide-*co*-glycolide), PVA—polyvinyl alcohol.

**Table 2:** Polymer matrices for dental topical applications produced by freeze-casting.

Type of Carrier	Polymers/ Excipients	Processing Parameters	API/ Application/ Indication	Ref.
Sponge	SA	Freezing time/temperature: 12 h/−20 °C Pressure/temperature: 0.01 atm/−60 °C	Tranexamic acid	[49]
Sponge	GG	Freezing temperature: −20 °C	Dental filling	[50]
Matrix	Gelatin	Freezing temperature: −15 °C Pressure: 9 × 10^−2^ to 1.3 × 10^−1^ mBar	Metronidazole/intra-pocket application	[51]
Wafers	SA, CMC	-	Paracetamol/application to mucosal surfaces, including wounds	[18]
Wafers	Karaya gum, guar gum, SA, xanthan gum	-	Chlorhexidine digluconate release against *Pseudomonas aeruginosa*	[52]
Wafers	CS	-	Protein drug delivery via the buccal mucosa	[53]
Wafers	CS, HPMC, glycerin, acetic acid solution	Monolayer wafer Freezing temperature: −20 °C overnight before freeze-drying Freeze-dried for 24 h in the following conditions: collector temperature: −80 °C; vacuum: 0.1 mBar Bilayer wafer First layer was frozen at −20 °C and before the second layer, an additional overnight freezing step was applied before the freeze-drying cycle	Cefuroxime axetil	[19]

CMC—sodium carboxymethyl cellulose, CS—chitosan, GG—gellan gum, HPMC—hydroxypropyl methylcellulose, SA—sodium alginate.

**Table 3:** Electrospinning parameters.

Processing Parameters	Solution Parameters	Ambient Parameters
Applied voltage Distance from needle tip to collector Needle inner diameter Solution flow rate Collector type	Solution viscosity Polymer concentration Polymer molecular weight Polymer solubility Conductivity Surface tension Solvent volatility	Air humidity Ambient temperature

**Table 4:** Applied electrospinning parameters.

Parameters	Values
Temperature	°C
Needle gauge	G
Distance between needle and collector	cm
Flow rate range	mL/h
High voltage range	kV

**Table 5:** Polymer matrices for dental topical applications produced by electrospinning.

Structure/ Carrier/ Morphology	Polymers/ Excipients/ Solvent	Processing Parameters	API and/or Application/ Activity/ Indication	Ref.
Nanofibers	Eudragit L, Eudragit S/ethanol	Flow rate: 0.5–2 mL/h	Ketoprofen	[61]
Core-sheath nanofibers	PVA, CS/acetic acid	Flow rate: 10 µL min^−1^/5 µL min^−1^ Distance: 5 cm Voltage: 25 kV Needle diameter: 1.02 mm/0.65 mm	Tetracycline hydrochloride	[62]
Nanofibrous membranes	PCL, ZnO, hexafluoroisopropanol	Needle diameter: 20 G Distance: 20 cm Voltage: 18 kV Flow rate: 2 mL h^−1^	Osteogenic and antibacterial/guided tissue regeneration	[63]
Nanofibers	PLGA, collagen/hexafluoroisopropanol	-	Lidocaine/ epinephrine	[64]
Electrospinning-based nanofibers/mucoadhesive two-layer patch	Rivelin^®^	-	Mucosal diseases clobetasol propionate	[22]
Double-layer mucoadhesive buccal film	CS, PVA, acetone/acetic acid	Distance: 20 cm Flow rate: 0.5–2 mL h^−1^ Voltage: 15 kV	Neem extract	[33]
Nanofibrous scaffolds	Gelatin, CS, SA, acetate buffer, citric acid	Voltage: 28 kV Distance: 15 cm Flow rate: 0.794 mL h^−1^	Restoring tissue integrity and treating bacterial infections	[65]
Electrospinning bilayer patch	PVP, Eudragit, polyethylene oxide, PCL, DCM, DMF	Voltage: 17 kV Flow rate: 1–5 mL h^−1^ Distance: 19 cm	Clobetasol propionate	[66]
Electrospun dual-layer patch delivery	PVP, Eudragit RS100, PEO 400 kDa, PCL DCM/DMF/ACN, ethanol	Solvent: 97% ethanol Voltage: 19 kV Distance: 14 cm Flow rate: 1.5 mL h^−1^	Electrospun patch delivery Lidocaine	[67]

ACN—acetonitrile, CS—chitosan, DCM—dichloromethane, DMF—dimethylformamide, GG—gellan gum, HPMC—hydroxypropyl methylcellulose, PEO—polyethylene oxide, PVP—polyvinylpyrrolidone, PC—polycaprolactone, PLGA—poly(D,L-lactide-co-glycolide), PVA—polyvinyl alcohol, SA—sodium alginate.

**Table 6:** Polymer matrices for dental topical applications produced by 3D printing.

Type of Carrier/API/ 3D Printing Method	Polymers/ Excipients	Processing Parameters	Application/ Activity/Indication	Ref.
3D-printed tooth caps with fluoride/FDM	PCL, PVA or PEG	1/NaF-loaded (10 wt%) composite filaments 1.75 ± 0.15 mm were produced by hot-melt extrusion (HME) 2/PCL/PVA and PCL/PEG filaments produced using FMD (nozzle temperatures set at 145 and 100 °C, the bed heated at 40 and 30 °C, respectively)	Sustained localized release of fluoride from personalized 3D-printed mouthguards at the device–enamel interface can improve the incorporation of fluoride in the tooth matrix and prevent lesion progression	[77]
Catechin-based film/SSE	HPMC/mannitol, glycerol, ethanol/water, Tween 80	1/Preparation of hydrogel-based printer material/ink 2/Extrusion parameters: printer syringe—27 G nozzle, air pressure through pump—20–70 kPa, the printed hydrogel was dried at room temperature and stored in a desiccator (air drying—AD); alternatively, the hydrogel was frozen at −80 °C and freeze-dried using an FD1000 freeze-dryer (freeze-drying—FD)	Oral cavity, including aphthous stomatitis and oral mucositis ulcers, catechins are flavonoids, and polyphenols exhibit antioxidant, anti-inflammatory, anti-cancer and anti-hypertensive effects	[78]
Multilayer film containing an ibuprofen/lidocaine ionic liquid/pressure-assisted microsyringe (PAM) printing	HPMC supporting layer, mannitol, water/Eudragit polymer layer (L100, EPO or RSPO), acetone, active substances	1/Preparation of hydrogel-based printer material/ink for Layer 1 Printing parameters of the 1st support layer: printer syringe—27 G nozzle, air pressure through pump—20–70 kPa, lyophilization at −80 °C 2/Preparation of printer material/ink for Layer 2 Printing parameters of Layer 2 applied to Layer 1: printing speed—10 mm/s; vertical shell 1, fill density—100%, multilayer film drying	Oral mucositis caused by radiation therapy and chemotherapy	[79]
Apigenin-loaded mucoadhesive oral film/SSE	HPMC, carbopol, poloxamer/ethanol	1/Preparation of hydrogel-based printer material/ink 2/Extrusion parameters: printer syringe—27 G nozzle, air pressure through pump—90 kPa, the printed hydrogel was dried at room temperature	Oral leucoplakia, including on the tongue	[80]

FDM—fused deposition modelling, HPMC—hydroxypropyl methylcellulose, PCL—polycaprolactone, PEG—polyethylene glycol, PVA—polyvinyl alcohol.

**Table 7:** Example of interpretation of diffusional release mechanisms from polymeric films [95].

Release Exponent (n)	Drug Transport Mechanism	Rate as a Function of Time
0.5	Fickian diffusion	t^−0.5^
0.45 < n = 0.89	Non-Fickian transport	t^n−1^
0.89	Case II transport	Zero-order release
Higher than 0.89	Super case II transport	t^n−1^

**Table 8:** Stability test of storage conditions for drug product [96,97].

Storage Condition	Stability Test Method	ICH Test Temperature/ Humidity/Period in Months
Room temperature	Long term	25 ± 2 °C/60 ± 5% RH/12
	Intermediate	30 ± 2 °C/65 ± 5% RH/6
	Accelerated	40 ± 2 °C/75 ± 5% RH/6

**Table 10:** Advantages and disadvantages of preparing polymeric carriers methods.

Technology	Advantages	Disadvantages/Challenges	Potential Solutions
Solvent-casting method (SCM)	- for uniform thin clear film - obtained film is flexible and high plasticity - method does not require specialized equipment for preparation	- polymer and additives should be soluble in a volatile solvent or water - possible residue toxic solvent in dry film - possible to inadvertently introduce air bubbles in mixture - limited possibility of controlling parameters during drying - method not adequate for thermosensitive substances - possible decrease in film flexibility during storage	- use centrifugation, vacuum or sonication for air removal from formulation - use method for content residual solvent evaluation, i.e., gas chromatographic (GC) - use packing container for moisture control and selection - use oven, dryer or hotplate and control drying parameter (time, temperature, moisture)
Freeze casting method (LM)	- possible to obtain products with expanded specific surface - method adequate for thermosensitive substances - product morphology can be adjusted by selection of excipients and process parameters - possible to control all parameters throughout the entire process	- long preparation process - multi-step process - method requires specialized equipment for preparation - high cost	- requires optimization of the formulation freezing stage (speed, temperature), which translates into pore size and an increase in the homogeneity of the resulting matrix - in pre-drying (sublimation) stage, the drying temperature determined should not exceed the melting point or glass-transition temperature of the resulting crystallites or amorphous forms, to not reduce the viscosity of the molded matrix and rigidity of its structure while limiting the formation of internal cracks, damage or kinks
Electrospinning method	- high surface area to volume ratio of nanofiber membrane - possible to obtain high-porosity product - possible to use electrospinning concept to spin nanofibrous membranes at industrial scale	- many parameters for optimization in method - obtaining different pore sizes - jet instability during process - use of organic solvents during process - obtaining different fiber thicknesses	- development of new approaches exploiting the use of greener and biocompatible solvents, for instance, water, avoiding the use of organic solvents; alternatively, use of melt electrospinning
3D printing method: fused deposition modelling (FDM)	- possible to obtain design of any shape and size for the carrier	- requires previous filament fabrication - physicochemical properties of the filament determine its printability - high-temperature process (possible thermal degradation of drug and excipients) - accessible devices are suitable for small-scale productions and print-on-demand products, but are not yet efficient enough for the production of pharmaceutical forms at an industrial rate - low drug loading	- analysis required of interactions occurring between API and the excipients used in FDM before filament fabrication - obtaining filament with appropriate diameter (not too small), which limits difficulties in manufacturing
3D printing method: semi-solid extrusion (SSE)	- low temperature process (suitable for thermolabile drugs) - high drug loading - low cost and fast production	- requires suitable viscosity of semisolids - requires postprocessing (e.g., drying)	
